# [Expression of Concern] Visfatin/PBEF/Nampt induces EMMPRIN and MMP-9 production in macrophages via the NAMPT-MAPK (p38, ERK1/2)-NF-κB signaling pathway

**DOI:** 10.3892/ijmm.2026.5794

**Published:** 2026-03-12

**Authors:** Yuqi Fan, Shu Meng, Yue Wang, Jiatian Cao, Changqian Wang

Int J Mol Med 27: 607-615, 2011; DOI: 10.3892/ijmm.2011.621

Following the publication of the above paper, a concerned reader has drawn the Editor's attention to the fact that, regarding the western blot data shown in Fig. 4A and E on p. 612, the p-p38 blots featured in these figure parts were strikingly similar, although the blots were rotated through 180° relative to each other. Upon investigating the data in this paper independently in the Editorial Office, it also came to light that β-actin control blots featured in Fig. 1E, and p-p38 blots featured in Fig. 5I, subsequently appeared in another paper written by the same research group in an article published in the journal *PLoS One.* Finally, control western blots appeared to have been re-used in Fig. 4A and C, although the experimental conditions reported for the western blots in these figure parts were different.

The authors have been contacted by the Editorial Office to offer an explanation for these apparent anomalies in the presentation of the data in this paper, and we are awaiting their response. Owing to the fact that the Editorial Office has been made aware of potential issues surrounding the scientific integrity of this paper, we are issuing an Expression of Concern to notify readers of these potential problems while the Editorial Office continues to investigate this matter further.

